# Tangential volumetric modulated arc therapy reduces cardiac and contralateral low-dose exposure compared with continuous volumetric modulated arc therapy in postoperative breast radiotherapy

**DOI:** 10.1016/j.phro.2026.101115

**Published:** 2026-11-02

**Authors:** Anne Caroline Knöchelmann, Mirko Fischer, Kathrin Burchardt, Daniela Meinecke, Jan-Niklas Becker

**Affiliations:** Department of Radiotherapy, Hannover Medical School, Carl-Neuberg-Strasse 1, Hannover, 30625, Germany

**Keywords:** Breast cancer, Postoperative breast radiotherapy, Tangential volumetric modulated arc therapy, Cardiac sparing, Low-dose bath, Contralateral breast dose, Secondary cancer risk, Organ-equivalent dose

## Abstract

**Background and purpose:**

Tangential volumetric modulated arc therapy may combine rotational conformality with tangential beam geometry. We compared tangential with continuous-arc volumetric modulated arc therapy for postoperative breast radiotherapy with deliberately matched target coverage, focusing on cardiac and contralateral low-dose exposure.

**Material and methods:**

Fifty-four patients were included in this paired planning study. For each patient, the clinically delivered tangential plan and a matched continuous-arc plan were evaluated. Target coverage was matched by clinical-target-volume D95% and planning-target-volume D2%. Dose-volume endpoints, low-dose area-under-the-curve metrics, delta dose-volume histograms, subgroup interactions, and mechanistic organ-equivalent-dose surrogates were evaluated.

**Results:**

Target coverage and homogeneity were comparable, while conformity was higher with tangential planning (*p* = 0.006). Tangential planning reduced mean heart dose from 1.34 to 1.17 Gy (*p* < 0.001), contralateral lung mean dose from 0.94 to 0.57 Gy, and contralateral breast mean dose from 1.16 to 0.76 Gy (both *p* < 0.001). Mechanistic organ-equivalent-dose surrogates were reduced by 37.0% for the contralateral lung and 32.7% for the contralateral breast. Exploratory interaction analyses did not identify statistically significant interactions by laterality, deep-inspiration breath hold, simultaneous integrated boost, or regional nodal irradiation.

**Conclusions:**

In a clinically heterogeneous paired cohort that included boost irradiation, regional nodal irradiation, and deep-inspiration breath hold, tangential volumetric modulated arc therapy reduced cardiac and contralateral low-dose exposure while preserving target coverage.

## Highlights

• Tangential arc therapy reduced cardiac low-dose exposure.
• Contralateral breast and lung low-dose exposure were lower.
• Target coverage was deliberately matched between planning techniques.

## Graphical abstract

**Figure:**
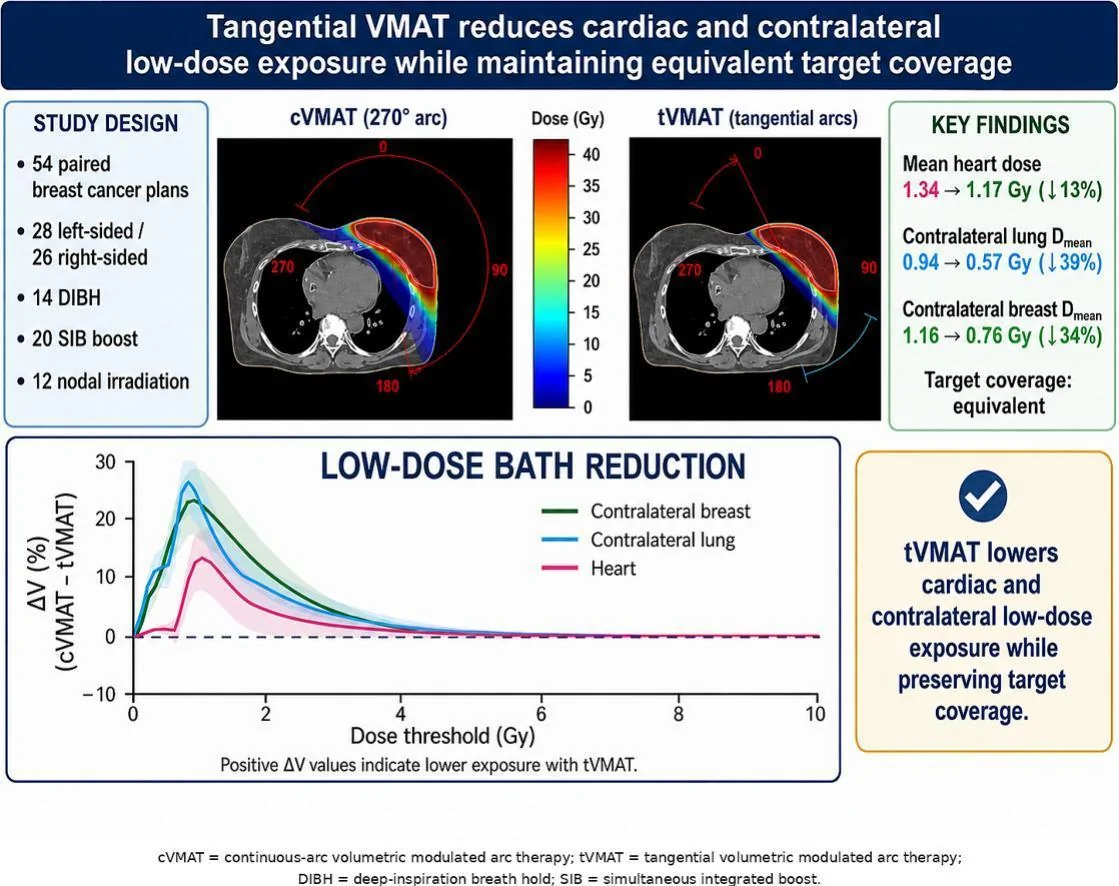

## 1 Introduction

Postoperative radiotherapy is an established component of breast-conserving treatment and postmastectomy management in selected patients, reducing locoregional recurrence and improving long-term breast cancer outcomes [1], [2], [3]. Because many patients have prolonged survival after early breast cancer, contemporary treatment planning increasingly aims not only to maintain target coverage but also to reduce avoidable exposure of normal tissues. The heart, coronary arteries, lungs, and contralateral breast are particularly relevant organs of interest because radiation exposure may contribute to late cardiac morbidity, pulmonary toxicity, and second primary malignancies [4], [5], [6], [7], [8], [9], [10], [11].

Volumetric modulated arc therapy can improve conformity and planning flexibility, especially for complex anatomy, simultaneous integrated boost, or regional nodal irradiation. However, broad rotational arc arrangements may expose larger volumes of healthy tissue to low doses. This trade-off is clinically relevant for the contralateral breast and contralateral lung, where high-dose exposure is usually low, but the integral low-dose bath may increase with rotational beam delivery [9], [10], [11], [12], [13], [14], [15]. Conversely, tangential partial-arc volumetric modulated arc therapy restricts beam entry to tangential sectors while retaining inverse optimization and rotational modulation [16], [17], [18], [19], [20].

Existing planning studies have mostly focused on selected left-sided cohorts or simplified target definitions. Less is known about tangential arc planning in a real-world postoperative cohort including left- and right-sided cases, deep-inspiration breath hold, tumour-bed boost, and regional nodal irradiation. In addition, conventional point metrics such as mean dose or individual Vx values may not fully describe whether the entire low-dose distribution is shifted. We therefore performed a paired planning study with intentionally matched target coverage and analysed delta dose-volume histograms, integrated low-dose area-under-the-curve metrics, exploratory subgroup interactions, and mechanistic organ-equivalent-dose surrogates. The study incorporated a within-patient design, deliberate target matching, clinically heterogeneous target definitions, and population-level characterization of the low-dose bath. We hypothesised that tangential arc planning would reduce cardiac and contralateral low-dose exposure while preserving target comparability.

## 2 Materials and methods

### 2.1 Study design and patients

This single-institution paired dosimetric planning study included 54 consecutive patients treated between January and December 2025 with postoperative breast radiotherapy using tangential volumetric modulated arc therapy. For each patient, a matched continuous-arc volumetric modulated arc therapy plan was generated retrospectively on the same planning computed tomography data set. Patients treated after breast-conserving surgery or mastectomy were eligible and were included irrespective of laterality, tumour-bed boost, regional nodal irradiation, or use of deep-inspiration breath hold. Patients treated with three-dimensional conformal radiotherapy or clinical continuous-arc volumetric modulated arc therapy were not included.

All patients underwent computed tomography simulation with 3-mm slice thickness in the supine position on a breast board with both arms above the head. Deep-inspiration breath hold was used when free-breathing planning did not meet institutional heart constraints. The study was approved by the Ethics Committee of Hannover Medical School on 15 September 2025 (approval number 11976_BO-K-2025) and was conducted in accordance with the Declaration of Helsinki.

### 2.2 Target volumes, organs of interest, and prescription

Target volumes were delineated by experienced radiation oncologists. The clinical target volume included the whole breast or chest wall as clinically indicated. Axillary or supraclavicular lymph node regions were included when regional nodal irradiation was indicated; no patient received internal mammary chain irradiation. Target-volume delineation and nodal indications followed breast radiotherapy guidance [21], [22]. The planning target volume was generated by expanding the clinical target volume by 6 mm and cropping it 3 mm from the body surface. Cardiac and thoracic organs of interest included the heart, left anterior descending coronary artery, right coronary artery, left ventricle, right ventricle, ipsilateral lung, contralateral lung, and contralateral breast. Cardiac substructures were delineated according to the atlas by Feng et al. [23].

The standard prescription was 40.05 Gy in 15 fractions [24], [25]. Selected patients received partial-breast dose reduction analogous to the IMPORT LOW concept, with 36.0 Gy to the whole breast and 40.05 Gy to the tumour bed in 15 fractions [26]. Twenty patients received a simultaneous integrated boost to 45.0 Gy in 15 fractions, reflecting contemporary hypofractionated boost concepts [27]. Twelve patients received regional nodal irradiation.

### 2.3 Treatment planning and target matching

All plans were generated in Monaco (Elekta, Stockholm, Sweden) and evaluated according to the principles of ICRU Report 83 [28]. The continuous-arc technique used a broad rotational geometry of approximately 270°. The tangential technique used two partial arcs of approximately 40–50° each, designed to preserve tangential beam entry while limiting unnecessary passage through contralateral thoracic tissue.

A central methodological feature of this study was deliberate target matching. For every paired comparison, the dose to 95% of the clinical target volume and the dose to 2% of the planning target volume were required to differ by no more than 0.3 Gy between plans. This constraint was introduced to ensure that differences in normal-tissue exposure were not explained by target undercoverage or relaxed hotspot control. Homogeneity and conformity indices were reported as planning-quality metrics and are defined below.

### 2.4 Dose-volume endpoints and low-dose metrics

Cumulative dose-volume histograms were exported for all relevant targets and organs of interest. Mean dose was calculated as the integral of the cumulative dose-volume histogram divided by the total structure volume. Dose-to-volume and volume-to-dose parameters were derived using inverse cumulative dose-volume interpolation. The low-dose bath was quantified using the area under the cumulative dose-volume curve between 0 and 5 Gy and between 0 and 10 Gy. Delta dose-volume histograms were calculated as the volume receiving at least a given dose threshold in the continuous-arc plan minus the corresponding volume in the tangential plan. Positive delta values therefore indicate lower exposure with tangential planning.

Mean heart dose was translated into an excess relative risk surrogate for major coronary events using the linear Darby model, excess relative risk = 0.074 × mean heart dose in Gy [5]. This transformation was used only to compare plans and was not intended as individualized clinical risk prediction.

### 2.5 Statistical analysis

Continuous variables are reported as mean ± standard deviation unless stated otherwise. Paired differences were defined as tangential plan minus continuous-arc plan; negative values therefore indicate lower exposure with tangential planning. Primary dosimetric comparisons used two-sided Wilcoxon signed-rank tests. To account for multiple comparisons, false-discovery-rate-adjusted q values were calculated using the Benjamini-Hochberg procedure. Exploratory interaction analyses assessed whether paired differences differed by laterality, deep-inspiration breath hold, simultaneous integrated boost, or regional nodal irradiation using two-sided Mann-Whitney *U* tests. These interaction analyses were considered hypothesis-generating and were not used to claim subgroup-specific superiority. Statistical analyses were performed in Python (Python Software Foundation, Wilmington, DE, USA) using SciPy and pandas.

Homogeneity index (HI) quantified target-dose uniformity and was calculated according to Eq. (1), where D2% and D95% are the doses received by 2% and 95% of the planning target volume, respectively.

(1) $$\mathrm{HI}=\frac{D_{2\%}}{D_{95\%}}$$

Conformity index (CI) was calculated according to Eq. (2), where VPTV,ref. is the part of the planning target volume covered by the reference dose, VPTV is the planning target volume, and Vref is the total volume covered by the reference dose.

(2) $$\mathrm{CI}=\frac{{\left(V_{\mathrm{PTV},\mathrm{ref}}\right)}^{2}}{V_{\mathrm{PTV}}\times V_{\mathrm{ref}}}$$

For the exploratory second-cancer surrogate analysis, cumulative dose-volume histograms of the contralateral breast and contralateral lung were converted to differential bins by successive volume differences. Organ-equivalent dose (OED) was calculated according to Eq. (3), where VT is the total organ volume, Vi is the differential volume in dose bin i, Di is the midpoint dose of differential bin i, and RED(Di) is the risk-equivalent dose. Eq. (3) is a linear volume-weighted aggregation of the bin-specific RED contributions; the underlying dose-response function itself was modelled with the non-linear full mechanistic RED model rather than with RED = D [29], [30].

(3) $$\mathrm{OED}=\frac{1}{V_{T}}\sum V_{i}\cdot \mathrm{RED}\left(D_{i}\right)$$

For carcinoma induction, risk-equivalent dose was calculated using the full mechanistic Schneider model according to Eq. (4). This formulation combines dose-dependent cell killing with repopulation and repair during fractionated irradiation [29], [30].

(4) $$\mathrm{RED}\left(D\right)=\frac{e^{-\alpha^{\prime }D}}{\alpha^{\prime }R}\;\left[1-2R+R^{2}\;e^{\alpha^{\prime }D}-{\left(1-R\right)}^{2}\;e^{\frac{-\alpha^{\prime }\mathrm{RD}}{1-R}}\right]$$

Fractionation was incorporated for each differential dose bin according to Eq. (5), where *n* = 15 fractions and α/β = 3 Gy. The full-mechanistic parameterization used α = 0.044 Gy^−1^ and *R* = 0.15 for female breast and α = 0.042 Gy^−1^ and *R* = 0.83 for lung, consistent with Schneider-based breast-radiotherapy risk modelling [15], [30]. OED was expressed in Gy and used as a comparative planning surrogate, not as an estimate of absolute second-cancer probability.

(5) $$\alpha_{i}^{\prime }=\alpha +\beta d_{i}=\alpha +\beta \;\frac{D_{i}}{n}$$

For Fig. 1B only, the mean paired mechanistic OED reduction was multiplied by the illustrative weighting factor in Eq. (6), where a denotes age in years, using 40 years as the reference and 85 years as an upper time horizon. The index was not used for hypothesis testing or absolute risk prediction; its linear age dependence is imposed by definition.

(6) $${\mathrm{\Delta OED}}_{\mathrm{age}}\left(a\right)=\left[{\mathrm{OED}}_{\mathrm{cVMAT}}-{\mathrm{OED}}_{\mathrm{tVMAT}}\right]\times \frac{85-a}{85-40}$$

**Fig. 1:**
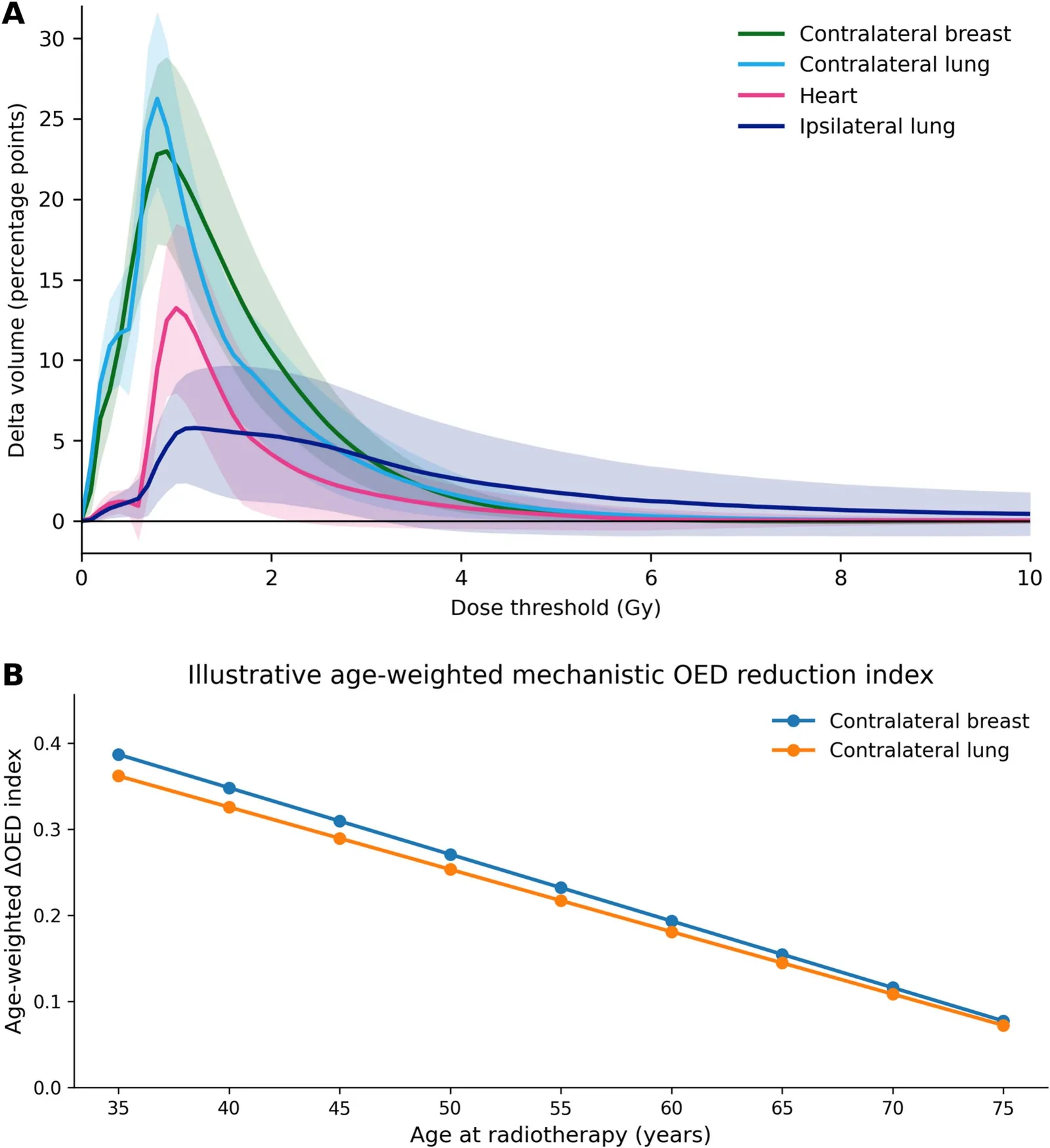
(A) Population delta dose-volume histogram curves. Positive values indicate lower volume exposure with tangential planning; shaded regions represent pointwise 95% confidence intervals around the mean delta-volume curve. (B) Illustrative age-weighted mechanistic organ-equivalent-dose reduction index for contralateral breast and contralateral lung. The linear age dependence is imposed by the weighting factor in Eq. (6); the index is not an estimate of absolute or age-specific second-cancer risk.

## 3 Results

The cohort included 54 patients. Twenty-eight patients had left-sided irradiation and 26 had right-sided irradiation. Deep-inspiration breath hold was used in 14 patients. Twenty patients received simultaneous integrated boost irradiation, and 12 patients received regional nodal irradiation. Breast-conserving surgery was performed in 51 patients and mastectomy in three patients; all 54 patients, including the three postmastectomy cases, were included in the paired dosimetric analyses (Table 1).

**Table 1:** Clinical and treatment characteristics. DIBH, deep-inspiration breath hold.

Characteristic	Number of patients
Patients	54
Left-sided irradiation	28 (51.9%)
Right-sided irradiation	26 (48.1%)
Deep-inspiration breath hold	14 (25.9%)
Free breathing	40 (74.1%)
Simultaneous integrated boost	20 (37.0%)
Regional nodal irradiation	12 (22.2%)
Breast-conserving surgery	51 (94.4%)
Mastectomy	3 (5.6%)

The prespecified target-matching criteria were fulfilled. The homogeneity index did not differ significantly between techniques, whereas the conformity index was higher with tangential planning (0.55 ± 0.12 vs 0.51 ± 0.13; *p* = 0.006). Tangential planning required more monitor units (632.6 +/− 99.0 vs 570.9 +/− 89.3; *p* < 0.001) and longer beam-on time (114.8 +/− 15.9 s vs 88.1 +/− 13.9 s; *p* < 0.001) (Table 2).

**Table 2:** Target comparability and treatment efficiency. Values are mean +/− standard deviation unless stated otherwise.

Parameter	Continuous arc	Tangential arcs	Difference	*p* value
Homogeneity index, planning target volume	1.17 +/− 0.07	1.17 +/− 0.08	−0.001	0.135
Conformity index, planning target volume	0.51 +/− 0.13	0.55 +/− 0.12	0.037	0.006
Monitor units	570.9 +/− 89.3	632.6 +/− 99.0	61.7	< 0.001
Beam-on time (s)	88.1 +/− 13.9	114.8 +/− 15.9	26.8	< 0.001

Cardiac results are shown in Table 3. Mean heart dose was lower with tangential planning (1.17 +/− 0.52 Gy vs 1.34 +/− 0.67 Gy; paired difference − 0.17 Gy, 95% confidence interval − 0.28 to −0.06; *p* < 0.001, q < 0.001). Heart volume receiving at least 1 Gy and 2 Gy, heart area under the curve from 0 to 5 Gy, and left anterior descending coronary artery volume receiving at least 1 Gy were also lower with tangential planning. Using the Darby model, the mean-heart-dose difference corresponded to an estimated excess-relative-risk reduction of 1.24 percentage points.

**Table 3:** Cardiac dose-volume endpoints. Differences are tangential arcs minus continuous arc.

Structure/endpoint	Continuous arc	Tangential arcs	Difference	95% CI	p value	q value
Heart mean dose (Gy)	1.34 +/− 0.67	1.17 +/− 0.52	−0.17	−0.28 to −0.06	< 0.001	< 0.001
Heart D1cc (Gy)	5.80 +/− 4.67	5.53 +/− 4.44	−0.27	−1.02 to 0.40	0.894	0.916
Heart V1 (%)	52.39 +/− 27.43	39.35 +/− 24.41	−13.04	−18.18 to −8.01	< 0.001	< 0.001
Heart V2 (%)	14.11 +/− 20.79	10.01 +/− 16.79	−4.10	−7.52 to −0.33	0.004	0.010
Heart V5 (%)	1.56 +/− 3.74	1.20 +/− 2.47	−0.36	−1.29 to 0.49	0.714	0.770
Heart area under curve 0–5 Gy (% x Gy)	130.39 +/− 59.98	114.09 +/− 47.99	−16.30	−25.88 to −6.74	< 0.001	< 0.001
Left anterior descending artery mean dose (Gy)	2.22 +/− 1.62	2.07 +/− 1.68	−0.15	−0.37 to 0.09	< 0.001	0.001
Left anterior descending artery D0.01cc (Gy)	22.09 +/− 18.05	27.79 +/− 17.59	5.70	0.51 to 10.47	0.038	0.076
Left anterior descending artery V1 (%)	70.04 +/− 36.57	54.38 +/− 43.85	−15.66	−22.54 to −10.00	< 0.001	< 0.001
Left anterior descending artery V2 (%)	36.39 +/− 35.16	32.20 +/− 36.95	−4.18	−9.05 to 0.07	0.095	0.173

Pulmonary, contralateral breast, and integrated low-dose endpoints are shown in Table 4. Ipsilateral lung mean dose, V20, and V15 were comparable. In contrast, tangential planning reduced contralateral lung mean dose from 0.94 +/− 0.53 Gy to 0.57 +/− 0.27 Gy and reduced contralateral breast mean dose from 1.16 +/− 0.49 Gy to 0.76 +/− 0.41 Gy. Contralateral lung V1, V2, V3, and area under the curve from 0 to 5 Gy were lower with tangential planning. Contralateral breast D2cc, V1, V2, and area under the curve from 0 to 5 Gy were also lower with tangential planning.

**Table 4:** Pulmonary, contralateral breast, and integrated low-dose endpoints. Differences are tangential arcs minus continuous arc.

Structure/endpoint	Continuous arc	Tangential arcs	Difference	95% CI	p value	q value
Ipsilateral lung mean dose (Gy)	6.34 +/− 2.42	6.26 +/− 2.45	−0.08	−0.67 to 0.48	0.288	0.410
Ipsilateral lung V20 (%)	9.17 +/− 4.07	9.29 +/− 4.08	0.12	−0.74 to 0.89	0.901	0.920
Ipsilateral lung V15 (%)	12.61 +/− 4.75	12.43 +/− 4.66	−0.18	−1.12 to 0.66	0.411	0.502
Ipsilateral lung V3 (%)	46.53 +/− 15.12	42.67 +/− 13.38	−3.86	−7.67 to −0.50	0.097	0.175
Contralateral lung mean dose (Gy)	0.94 +/− 0.53	0.57 +/− 0.27	−0.37	−0.48 to −0.27	< 0.001	< 0.001
Contralateral lung V1 (%)	30.38 +/− 22.30	8.81 +/− 20.17	−21.56	−26.23 to −16.70	< 0.001	< 0.001
Contralateral lung V2 (%)	8.45 +/− 14.03	0.70 +/− 2.73	−7.76	−11.54 to −4.61	< 0.001	< 0.001
Contralateral lung V3 (%)	3.47 +/− 8.94	0.04 +/− 0.17	−3.43	−5.99 to −1.46	< 0.001	< 0.001
Contralateral lung area under curve 0–5 Gy (% x Gy)	93.56 +/− 50.05	57.48 +/− 26.58	−36.08	−46.34 to −26.90	< 0.001	< 0.001
Contralateral breast mean dose (Gy)	1.16 +/− 0.49	0.76 +/− 0.41	−0.40	−0.52 to −0.29	< 0.001	< 0.001
Contralateral breast D2cc (Gy)	4.01 +/− 1.73	2.33 +/− 1.71	−1.68	−2.10 to −1.27	< 0.001	< 0.001
Contralateral breast V1 (%)	41.00 +/− 24.76	18.91 +/− 22.17	−22.08	−27.91 to −15.71	< 0.001	< 0.001
Contralateral breast V2 (%)	14.16 +/− 16.01	3.66 +/− 10.74	−10.50	−14.90 to −6.47	< 0.001	< 0.001
Contralateral breast area under curve 0–5 Gy (% x Gy)	115.34 +/− 47.48	75.34 +/− 38.78	−40.00	−51.39 to −28.73	< 0.001	< 0.001

The full mechanistic OED analysis showed the same direction of effect as conventional dose-volume metrics. Contralateral breast OED decreased from 1.06 ± 0.41 Gy with continuous-arc planning to 0.72 ± 0.34 Gy with tangential planning (mean reduction 0.35 Gy, 32.7%; *p* < 0.001). Contralateral lung OED decreased from 0.88 ± 0.45 to 0.56 ± 0.24 Gy (mean reduction 0.33 Gy, 37.0%; *p* < 0.001). These OED values are comparative risk surrogates and not estimates of absolute second-cancer incidence.

Exploratory interaction analyses were non-significant for laterality, deep-inspiration breath hold, simultaneous integrated boost, and regional nodal irradiation (Table 5). For left- versus right-sided irradiation, mean heart dose changed from 1.47 to 1.33 versus 1.20 to 1.00 Gy (interaction *p* = 0.574); heart V1 from 55.49 to 46.94 versus 49.06 to 31.19% (*p* = 0.065); heart V2 from 18.52 to 14.93 versus 9.36 to 4.71% (*p* = 0.802); and heart AUC0–5 Gy from 141.02 to 128.27 versus 118.95 to 98.83%·Gy (*p* = 0.634). Contralateral low-dose AUC interactions were also non-significant (all *p* ≥ 0.101).

**Table 5:** Exploratory subgroup analyses of representative dosimetric endpoints. Δ denotes the mean paired reduction (continuous arc minus tangential arcs); positive values indicate lower exposure with tangential planning. Interaction *p* values compare paired differences between subgroups using Mann-Whitney *U* tests. DIBH, deep-inspiration breath hold; SIB, simultaneous integrated boost; RNI, regional nodal irradiation; pp., percentage points.

Subgroup	Endpoint	Subgroup A: mean Δ	Subgroup B: mean Δ	Interaction p
Laterality (left *n* = 28; right *n* = 26)	Heart mean dose (Gy)	Left 0.135	Right 0.202	0.574
	Contralateral lung mean dose (Gy)	Left 0.452	Right 0.280	0.373
	Contralateral breast mean dose (Gy)	Left 0.454	Right 0.347	0.295
	Ipsilateral lung V20 (pp)	Left −0.310	Right 0.089	0.431
DIBH (yes *n* = 14; no *n* = 40)	Heart mean dose (Gy)	Yes 0.269	No 0.131	0.338
	Contralateral lung mean dose (Gy)	Yes 0.531	No 0.313	0.121
	Contralateral breast mean dose (Gy)	Yes 0.486	No 0.373	0.348
	Ipsilateral lung V20 (pp)	Yes 0.011	No −0.163	0.141
SIB (yes *n* = 20; no *n* = 34)	Heart mean dose (Gy)	Yes 0.198	No 0.149	0.490
	Contralateral lung mean dose (Gy)	Yes 0.469	No 0.311	0.101
	Contralateral breast mean dose (Gy)	Yes 0.382	No 0.414	0.837
	Ipsilateral lung V20 (pp)	Yes −1.317	No 0.587	0.227
RNI (yes *n* = 12; no *n* = 42)	Heart mean dose (Gy)	Yes 0.333	No 0.120	0.257
	Contralateral lung mean dose (Gy)	Yes 0.549	No 0.318	0.180
	Contralateral breast mean dose (Gy)	Yes 0.406	No 0.401	0.685
	Ipsilateral lung V20 (pp)	Yes −0.184	No −0.099	0.827

Population delta dose-volume histograms showed that the separation between techniques was concentrated below approximately 3–5 Gy (Fig. 1A), with peak mean reductions at 0.9 Gy for the contralateral breast and 0.8 Gy for the contralateral lung. Fig. 1B displays the illustrative age-weighted mechanistic OED reduction index; its linear decline with scenario age is imposed by Eq. (6), not an observed age effect.

## 4 Discussion

This paired planning study showed that tangential volumetric modulated arc therapy using two partial arcs reduces cardiac and contralateral low-dose exposure compared with broad continuous-arc volumetric modulated arc therapy while maintaining matched target coverage. Its methodological contribution is the combination of strict target matching, population delta dose-volume analysis, integrated low-dose metrics, exploratory subgroup testing, and a mechanistic organ-equivalent-dose model, thereby extending the comparison beyond isolated point metrics.

The observed pattern is physically coherent. Restricting arc geometry to tangential sectors did not materially change the target-adjacent high-dose region of the ipsilateral lung, as reflected by similar ipsilateral lung V20. Instead, it reduced the number of beam directions traversing the heart and contralateral thoracic tissues. This explains why the largest separation was seen in low-dose cardiac and contralateral structures rather than in ipsilateral high-dose lung parameters.

The cardiac findings are relevant because even small reductions in mean heart dose may matter in a curative population with long life expectancy. Darby et al. reported a linear increase in major coronary events with mean heart dose [5], and more recent work has emphasized the additional relevance of cardiac substructures, including coronary arteries [7], [8]. In the present study, the absolute mean-heart-dose difference was modest, but it was accompanied by reductions in heart V1, heart V2, heart low-dose area under the curve, and left anterior descending coronary artery V1. These findings suggest that tangential planning modifies the low-dose cardiac bath rather than merely shifting a single summary parameter. However, tangential arc planning was associated with a higher near-maximum dose to the left anterior descending coronary artery (LAD D0.01cc), highlighting a potential trade-off between reduced overall cardiac exposure and localized coronary artery dose.

The results should not be interpreted as a replacement for deep-inspiration breath hold. Breath hold and tangential arc restriction address different mechanisms: breath hold improves anatomical separation between the heart and chest wall, whereas tangential arc restriction reduces beam entry through non-target thoracic tissues [31], [32], [33]. No statistically significant interaction was identified for breath hold, laterality, simultaneous integrated boost, or regional nodal irradiation. Because these analyses were underpowered for interaction testing, absence of statistical interaction should not be interpreted as evidence of equivalent effects across subgroups.

The most pronounced dosimetric effect was observed for contralateral tissues. Contralateral breast and lung mean dose and integrated low-dose exposure were lower with tangential planning, whereas ipsilateral lung high-dose parameters remained comparable, indicating no relevant increase in high-dose lung exposure associated with pneumonitis risk [34], [35], [36]. Modern rotational techniques may increase low-dose exposure of healthy tissue, and stochastic late effects are generally modelled without a strict threshold in the low-dose range [9], [10], [11], [37], [38], [39], [40], [41], [42]. These dosimetric findings do not prove a reduction in second malignancies.

The mechanistic OED analysis provides a model-based interpretation of the low-dose reduction. OED was developed to compare inhomogeneous organ dose distributions in terms of radiation-induced cancer risk, and the full mechanistic RED model incorporates cell killing and repopulation/repair during fractionated treatment [29], [30]. Using organ-specific Schneider parameters, tangential planning reduced OED by approximately one third for both contralateral breast and lung. Similar approaches have been applied in breast-radiotherapy planning studies [15], [37], [41]. OED remains a model-dependent comparative surrogate and should not be interpreted as an individual cancer probability.

Fig. 1B is an illustrative sensitivity display. Its linear age dependence is imposed by the weighting factor and is not evidence of greater clinical benefit at younger ages. It does not estimate lifetime attributable risk or absolute cancer probability.

A practical trade-off was treatment efficiency. Tangential planning required higher monitor units and longer beam-on time. In daily practice, the potential benefit of reducing cardiac and contralateral low-dose exposure must therefore be balanced against treatment-room time, intrafractional stability, breath-hold reproducibility, and departmental workflow. Nevertheless, the mean beam-on time difference was less than 30 s, suggesting that the efficiency penalty may be clinically acceptable when low-dose sparing is prioritized.

This study is limited by its single-institution planning design and dependence on contouring, optimization priorities, and planner experience. Clinical toxicity and second malignancies cannot be inferred from dosimetric data. Subgroup interaction analyses were exploratory and underpowered, particularly for deep-inspiration breath hold and regional nodal irradiation. The mechanistic OED model is parameter dependent and should be interpreted as a comparative surrogate rather than an individual risk prediction. Multicentre and prospective studies are needed to determine whether the dosimetric advantages translate into measurable long-term benefit.

In summary, tangential volumetric modulated arc therapy reduced cardiac and contralateral low-dose exposure compared with broad continuous-arc volumetric modulated arc therapy with matched target coverage. Target homogeneity was preserved and conformity improved, at the cost of higher monitor units and longer beam-on time. Exploratory subgroup analyses did not identify statistically significant interactions, and the mechanistic OED analysis supported the same direction of effect for contralateral breast and lung. Tangential partial-arc planning may therefore be useful when cardiac and contralateral low-dose sparing is prioritized.

## Declaration of generative artificial intelligence and assisted technologies in manuscript preparation

During the preparation of this work, the authors used ChatGPT for improving the clarity of the text. The authors reviewed and edited the output as needed and take full responsibility for the content of the published article.

## CRediT authorship contribution statement

**Anne Caroline Knöchelmann:** Writing – review & editing, Writing – original draft, Visualization, Validation, Supervision, Resources, Project administration, Investigation, Formal analysis, Data curation, Conceptualization. **Mirko Fischer:** Validation, Software, Resources, Data curation. **Kathrin Burchardt:** Writing – review & editing, Methodology, Data curation. **Daniela Meinecke:** Writing – review & editing, Validation, Resources, Data curation. **Jan-Niklas Becker:** Writing – review & editing, Writing – original draft, Visualization, Validation, Supervision, Software, Resources, Methodology, Formal analysis, Data curation, Conceptualization.

## Funding

This research received no specific grant from any funding agency in the public, commercial, or not-for-profit sectors.

## Declaration of competing interest

The authors declare that they have no competing financial interests or personal relationships that could have appeared to influence the work reported in this paper.

## Data Availability

The dosimetric data underlying this study may be made available by the corresponding author upon reasonable request, subject to institutional and data-protection requirements.
